# Social Influence and Uptake of Couples HIV Testing and Counselling in KwaZulu-Natal, South Africa

**DOI:** 10.1007/s10461-021-03435-1

**Published:** 2021-08-21

**Authors:** Matthew J. Johnson, Lynae A. Darbes, Victoria Hosegood, Mallory O. Johnson, Katherine Fritz, Thulani Ngubane, Heidi van Rooyen, Nuala McGrath

**Affiliations:** 1 MRC Lifecourse Epidemiology Unit, Southampton General Hospital, University of Southampton, Mailpoint 95, Tremona Road, Southampton, SO16 6YD UK; 2 NIHR ARC Wessex Data Science Hub, Faculty of Environmental & Life Sciences, University of Southampton, Southampton, UK; 3 Center for AIDS Prevention Studies, Division of Prevention Sciences, School of Medicine, University of California, San Francisco, San Francisco, CA USA; 4 Department of Health Behavior and Biological Sciences, Center for Sexuality and Health Disparities, University of Michigan School of Nursing, Ann Arbor, USA; 5 Department of Social Statistics and Demography, Faculty of Social, Human and Mathematical Sciences, University of Southampton, Southampton, UK; 6 Africa Health Research Institute, KwaZulu-Natal, South Africa; 7 International Center for Research on Women, Washington, DC USA; 8 Human Sciences Research Council, Durban, South Africa; 9 School of Clinical Medicine, Faculty of Health Sciences, University of the Witwatersrand, Johannesburg, South Africa; 10 School of Primary Care, Population Sciences and Medical Education, Faculty of Medicine, University of Southampton, Southampton, UK

**Keywords:** HIV prevention, Couples HIV testing and counselling, Social influence, Peer support, South Africa

## Abstract

Social influences may create a barrier to couples HIV testing and counselling (CHTC) uptake in sub-Saharan Africa. This secondary analysis of data collected in the ‘Uthando Lwethu’ randomised controlled trial used discrete-time survival models to evaluate the association between within-couple average ‘peer support’ score and uptake of CHTC by the end of nine months’ follow-up. Peer support was conceptualised by self-rated strength of agreement with two statements describing friendships outside of the primary partnership. Eighty-eight couples (26.9%) took up CHTC. Results tended towards a dichotomous trend in models adjusted only for trial arm, with uptake significantly less likely amongst couples in the higher of four peer support score categories (OR 0.34, 95% CI 0.18, 0.68 [7–10 points]; OR 0.53, 95% CI 0.28, 0.99 [≥ 11 points]). A similar trend remained in the final multivariable model, but was no longer significant (AOR 0.59, 95% CI 0.25, 1.42 [7–10 points]; AOR 0.88, 95% CI 0.36, 2.10 [≥ 11 points]). Accounting for social influences in the design of couples-focused interventions may increase their success.

## Introduction

Most HIV transmission in sub-Saharan Africa (SSA) occurs within marital or cohabiting couple relationships [1, 2]. Within-couple serodiscordancy is common [3, 4], but the risk of HIV transmission could still be reduced by adopting appropriate preventive behaviours [5, 6]. Knowledge of each partner’s HIV status could prompt couples to re-evaluate, and perhaps change, their behaviour [7], and has been associated with a decline in unprotected sex [8], particularly where serodiscordancy is known [9]. However, this knowledge has often been lacking in SSA [10–12], whether product of disinclination to seek or accept testing [13], a preference for inferring personal status from that of the partner [14, 15], or active choice not to disclose [16, 17]. Irrespective of the reason, as non-awareness of personal and/or partner HIV status is a barrier to engagement with prevention behaviours, there is an ongoing need to expand HIV testing and facilitate mutual disclosure amongst couples.

Historically, many models of HIV prevention have prioritised the internal beliefs and motivations of the individual as determining their propensity to adopt health behaviours, but this perspective may be unable to explain actions guided by thoughts and feelings about the partner, or unequal relationship power [18]. Indeed, the inherently dyadic dimension to HIV transmission has prompted calls for greater utilisation of couples-focused interventions that explicitly recognise, address and leverage characteristics of the relationship [18, 19], with such interventions consistently shown to encourage testing and other prevention behaviours more effectively than those targeted to individuals [20, 21]. Couples HIV testing and counselling (CHTC) has been shown to precipitate a sustained reduction in unprotected sex and partner concurrency [22], and is recommended by the World Health Organisation for its emphasis on mutual testing and disclosure, followed by the delivery of tailored counselling messages to address couple status and facilitate joint decision-making [23]. Nonetheless, uptake remains low [24], suggesting further barriers for couples who might otherwise seek or accept the intervention.

According to Lewis and colleagues’ interdependence model [25], couple-level behaviour change is shaped by partners’ ability to transition from a purely personal approach to health risks to one that assigns them greater meaning via direct association with the relationship, and their desire to see it continue. When partners are united in their assessment of risk and the approach to its management, they are more able to make decisions and act collaboratively. Nonetheless, the primary partnership remains just one of many interpersonal relationships experienced by an individual, each of which may shape their behaviour to some extent. Personal decision-making guided by anticipation of social reward or censure, or self-comparison with others, may lead some to align themselves with norms established and maintained by their structural social context [26]. In SSA, there is evidence to suggest that social influences may discourage testing and undermine HIV prevention efforts. Fear of stigmatisation, lost personal relationships or diminished social standing following a positive result or association with HIV services may result in testing delay or avoidance [13]. Aspiration to an idealised ‘masculine’ self-identity framed around presenting personal qualities of self-confidence, strength and invulnerability shapes normative male attitudes to health-seeking and risk behaviours in SSA [27, 28]. Male peer groups, in particular, may perpetuate harmful normative attitudes and behaviours amongst members, by causing some to avoid association with health services for fear of projecting an image of weakness [27, 28], or by providing social approval, encouragement and practical advice to support concurrent [29] or extramarital relationships [30, 31].

Much as directly addressing the relationship between partners has proven an effective HIV prevention strategy, other kinds of established social relationships have emerged as a viable platform for intervention in SSA. For example, invitations to CHTC were found to have greater chance of success when issued by family members or other social acquaintances rather than hitherto unknown agents [32, 33]. Furthermore, peer leader interventions have been associated with an increase in safer sex behaviours and improved knowledge and attitudes regarding HIV risk and transmission among urban working women [34], and with increased HIV testing and reduced inequitable gender norm attitudes among male peer networks [35]. Finally, follow-up after a peer group intervention revealed a broader increase in recent testing and improvement in attitudes to condom use across the study locality [36], suggesting that new ideas, information or behaviours perceived as valuable or beneficial may have further appeal to, and be adopted by, those not directly involved in the initial intervention.

Although recent qualitative research from Kenya has suggested that social influences can reinforce gender norms and inequalities within marriage, with negative implications for HIV transmission risk [37], most studies have examined their effect upon individuals only. To further explore their effect on couples, this secondary analysis of existing data collected as part of the Uthando Lwethu (‘Our Love’) randomised controlled trial evaluated the association between men’s, women’s and couples’ perceived access to peer support and the outcome of CHTC uptake, in the context of other demographic and relationship factors.

## Methods

### Primary Study

The Uthando Lwethu trial was an efficacious behavioural couples-focused intervention that significantly increased CHTC uptake in rural KwaZulu-Natal province, South Africa, under the hypothesis that couples with improved relationship quality following couples counselling would be more likely to take up CHTC together [38]. Heterosexual couples aged 18 to 50 years in a sexually active relationship of minimum six months’ duration were recruited from the Vulindlela sub-district of KwaZulu-Natal. Those with prior mutual disclosure or history of couples testing were excluded. Recruitment took place from March 2012 to August 2014, at which time the 15–49 age group accounted for more than half of the provincial population [39] and had HIV prevalence at 27.9% [40]. Overall, 332 couples were recruited and followed for nine months, during which time six broke up and sixteen were lost to follow-up. Immediately following randomisation all intervention and control couples were eligible for CHTC provided by study staff, and received text message reminders of its availability twice monthly. Partners received an initial health information group session together, after which intervention couples received a further single-sex group session and four couples counselling sessions designed to improve and promote relationship quality. Partners were separately, but simultaneously, interviewed face-to-face in isiZulu language by a gender-matched interviewer at four timepoints: baseline and 3, 6 and 9 months, with individual reports linked for analysis using a couple identifier. Responses were recorded using mobile phone data capture. Demographic and socio-economic information was collected at baseline only. Sexual, health and relationship behaviours and markers of relationship quality were collected at all timepoints.

### Ethics

Ethical approval for the primary study was obtained through the Committee on Human Research of the University of California, San Francisco, the Research Ethics Committee of the Human Sciences Research Council in South Africa, and London School of Hygiene and Tropical Medicine, UK. The study protocol is available at http://www.clinicaltrials.gov. Additional detail concerning intervention and study procedures have been published previously [41]. Ethical approval for this secondary data analysis was obtained from the Ethics Committee of the Faculty of Social, Human and Mathematical Sciences, University of Southampton.

### Independent Variable

Couples’ perceived access to peer support was the independent variable of interest and, for this analysis, was conceptualised by self-rated strength of agreement with two statements describing friendships outside of the primary partnership. The statements were adapted from a standardised scale measuring sense of autonomy within the relationship [42], and translated into isiZulu:

- ‘I have a supportive group of friends separate from my partner’ (‘friends’ score);
- ‘I have a close friend other than my partner’ (‘close friend’ score).

Agreement with each statement was separately scored on a nine-point scale, and scores were summed to generate a composite ‘peer support’ (PS) score per partner (maximum range 2 to 18 points). Within-couple mean scores were used as couple-level average (range 2 to 18 points) and female scores were subtracted from male scores to calculate couple-level difference (range − 16 to + 16 points), the latter considered a potential confounder.

### Outcome Variable

Although a binary outcome for CHTC uptake during follow-up was used for some analyses, time to uptake was also considered using discrete-time survival models. Couples were considered at risk from date of randomisation, and censored if they had not taken up CHTC by 9 months’ follow-up.

### Adjustment Variables

Recognising the possibility that the selected statements might also describe individuals engaging in outside relationships or activities as means of coping with an unsatisfactory primary partnership, measures of relationship satisfaction [43] and intimacy [42] were analysed as potential confounders. Each measure was adapted from a standardised scale: the former indicated by self-rating one interview item (‘In general, how satisfied are you with your relationship?’) on a six-point numeric scale [43]; the latter a composite measure using five interview items (for example, ‘I spend as much time with my partner as possible’), each on a nine-point numeric scale [42]. In each case couple-level average and difference scores were calculated and converted to categorical variables for analysis. Categorisations were derived following the same method as couple PS scores, outlined below.

As around 90% of study couples were unmarried relationship status was categorised by cohabitation, which was assumed if reported by at least one partner; 94% of within-couple reports were concordant; five couples without valid report were excluded from all analyses. Within-couple age difference, partnership length, employment status, receipt of state grant (including child support grants, disability grants and workman’s compensation), educational attainment (completion of secondary education, also called matric) and religion were also considered. Most variables were categorised using couple-level definitions, although male and female religion, for which around 65% of within-couple reports were discordant, were taken separately. Furthermore, as over 90% of within-couple partnership length reports differed by less than 1 year, the male report was used to represent the couple. Finally, owing to the significant intervention effect [38] all models were adjusted for trial arm; models with one additional explanatory variable are henceforth described as *bivariable*.

### Analysis

Exploratory random effects modelling was used to assess change in couple average PS score across the four survey timepoints (results not shown). As no statistically significant change was detected, all subsequent modelling treated scores and covariates as time-invariant and fixed at baseline.

Between-group comparisons on the dichotomised outcome used the chi-squared test for categorised characteristics of the study cohort, and the Mann–Whitney test for non-normally distributed male, female and couple PS score variables. All tests used reports at baseline.

Couple average, couple difference and individual partner PS scores were analysed as categorical variables rather than assuming a linear relationship with the outcome, with exploratory analysis used to determine the most appropriate categorisations. Histograms representing the distribution of each continuous variable were created, and two-way tables between categorised variables and the outcome were used to see how the proportion meeting the outcome varied across categories. Categories were specified in line with natural groupings suggested by the histogram, with an effort to balance sample size across categories, ensure parsimony in the final models and avoid introducing heterogeneity in the relationship with the outcome within categories. Binary categorisations were used for male (at ≥ 8 points) and female (at ≥ 10 points) partner scores, whereas four categories (at ≤ 4, 5 to 6, 7 to 10 and ≥ 11 points) were required for couple average and five categories (at ≤ − 7, − 6 to − 2, − 1 to + 1, + 2 to + 6 and ≥  + 7) for couple difference.

To compare the relative probability of the outcome across couple average score categories, survival estimates at nine months’ follow-up were calculated, using the log-rank test for significant differences [44]. Modelling time to outcome (in days) using a Cox proportional hazards specification was ruled out owing to failure to satisfy the proportional hazards assumption. Instead, discrete-time survival models (taking month as the unit of time) were used, expressing results as odds ratios. As the majority of events occurred early in the follow-up period the final four follow-up months were grouped into a single time unit.

Variables significant at the 10% level in bivariable discrete-time survival models were introduced to a multivariable model in descending order of significance, iteratively assessed using the likelihood ratio test and retained in the final model if significant at the 5% level. Lastly, to assess the effect of male and female partner PS scores on CHTC uptake, a further model was created replacing couple average and difference PS scores with both individual partner variables.

All statistical analyses used Stata SE Version 14.0 (StataCorp, College Station, TX, USA).

## Results

Of 327 couples, 88 (26.9%) took up CHTC by nine months’ follow-up. Unadjusted analysis indicated statistically significant differences by outcome for male PS score (χ^2^ = 5.40, df = 1, p = 0.020), female PS score (χ^2^ = 4.00, df = 1, p = 0.045), couple average PS score (χ^2^ = 10.87, df = 3, p = 0.012), cohabitation (χ^2^ = 9.70, df = 1, p = 0.002) and female-reported religion (χ^2^ = 8.35, df = 3, p = 0.039) (Table 1). Couples with CHTC uptake also had lower couple average (significant; z = 2.18, p = 0.030) and male partner (borderline; z = 1.92, p = 0.054) PS scores, and significantly lower couple average (z = 2.78, p = 0.005) and male partner (z = 2.39, p = 0.017) scores on the ‘friends’ component of the overall PS score (Table 2). Furthermore, time to uptake varied over different levels of couple average PS score (log-rank; χ^2^ = 11.81, df = 3, p = 0.008), with survival estimates indicating a higher likelihood and shorter time to uptake by nine months follow-up for couples with lower scores (Table 3).

**Table 1:** Baseline characteristics of couples included in analysis

Characteristic	No CHTC uptake n = 239 (73.1%)		CHTC uptake n = 88 (26.9%)		Test statistic^a^	df	p-value
	n	% (column)	n	% (column)			
Male peer support score							
7 points or less	96	40.2	48	54.6	5.40	1	0.020^b^
8 points or more	143	59.8	40	45.5			
Female peer support score							
9 points or less	128	53.6	58	65.9	4.00	1	0.045^b^
10 points or more	111	46.4	30	34.1			
Couple average peer support score							
4 points or less	50	20.9	26	29.6	10.87	3	0.012^b^
5–6 points	41	17.2	25	28.4			
7–10 points	72	30.1	16	18.2			
11 points or more	76	31.8	21	23.9			
Couple difference peer support score							
− 7 points or more (female higher)	49	20.5	18	20.5	2.71	4	0.607
− 6 to − 2 points	40	16.7	15	17.1			
− 1 to + 1 points	62	25.9	28	31.8			
+ 2 to + 6 points	41	17.2	16	18.2			
+ 7 points or more (male higher)	47	19.7	11	12.5			
Couple average satisfaction score							
5 points or less	37	15.5	8	9.1	2.21	1	0.137
6 points	202	84.5	80	90.9			
Couple difference satisfaction score							
0 points or less (equal, or female higher)	103	43.1	35	39.8	0.29	1	0.589
+ 1 point or more (male higher)	136	56.9	53	60.2			
Couple average intimacy score							
39 points or less	62	25.9	19	21.6	0.68	2	0.713
40–42 points	124	51.9	49	55.7			
43 points or more	53	22.2	20	22.7			
Couple difference intimacy score							
− 3 points or more (female higher)	76	31.8	20	22.7	6.21	2	0.045^b^
− 2 to + 2 points	60	25.1	34	38.6			
+ 3 points or more (male higher)	103	43.1	34	38.6			
Couple age difference (male minus female)							
− 2 years or more (female older)	36	15.1	8	9.1	6.19	3	0.103
− 1 to + 1 year	76	31.8	23	26.1			
+ 2 to + 5 years	75	31.4	40	45.5			
+ 6 years or more (male older)	52	21.8	17	19.3			
Partnership length							
Less than 2 years	40	16.7	16	18.2	5.45	3	0.141
2 to 4 years	119	49.8	33	37.5			
5 to 9 years	51	21.3	21	23.9			
10 years or more	29	12.1	18	20.5			
Cohabitation							
No	194	81.2	57	64.8	9.70	1	0.002^b^
Yes	45	18.8	31	35.2			
Employment status							
Male unemployed	148	61.9	61	69.3	1.56	2	0.458
Only male employed	69	28.9	21	23.9			
Both employed	22	9.2	6	6.8			
Receipt of state employment grant							
Neither partner receives grant	117	49.0	37	42.1	1.23	1	0.267
At least one partner receives grant	122	51.1	51	58.0			
Educational attainment							
Both have matric or higher	63	26.4	17	19.3	4.85	3	0.184
Only male has matric or higher	47	19.7	14	15.9			
Only female has matric or higher	51	21.3	17	19.3			
Both have incomplete secondary or lower	78	32.6	40	45.5			
Religion (female reported)							
None	18	7.5	16	18.2	8.35	3	0.039^b^
Christian	135	56.5	42	47.7			
Zionist	61	25.5	23	26.1			
Other	25	10.5	7	8.0			
Religion (male reported)							
None	86	36.0	40	45.5	6.37	3	0.095^c^
Christian	98	41.0	27	30.7			
Zionist	38	15.9	10	11.4			
Other	17	7.1	11	12.5			

^a^Chi-squared test statistic

^b^Significant at p < 0.05 level

^c^Significant at p < 0.10 level

**Table 2:** Distribution of male, female and couple peer support and component scores at baseline, by outcome

Variable	No CHTC uptake n = 239 (73.1%)	CHTC uptake n = 88 (26.9%)	Test statistic^a^	p-value
	Median (IQR)	Median (IQR)		
Peer support score				
Male	9.0 (2.0, 13.0)	5.0 (2.0, 11.0)	1.92	0.054^c^
Female	9.0 (3.0, 16.0)	8.5 (3.0, 10.0)	1.02	0.310
Couple average	9.0 (5.5, 12.0)	6.5 (3.0, 10.5)	2.18	0.030^b^
Couple difference (male minus female)	0.0 ( − 6.0, 5.0)	0.0 (− 4.5, 3.0)	0.42	0.672
‘Friends’ score				
Male	5.0 (1.0, 7.0)	2.0 (1.0, 7.0)	2.39	0.017^b^
Female	2.0 (1.0, 8.0)	2.0 (1.0, 8.0)	1.44	0.149
Couple average	4.5 (1.5, 6.5)	3.5 (1.5, 4.75)	2.78	0.005^b^
Couple difference (male minus female)	0.0 (− 1.0, 3.0)	0.0 (− 1.0, 1.0)	0.39	0.698
‘Close friend’ score				
Male	2.0 (1.0, 7.0)	2.0 (1.0, 7.0)	0.66	0.510
Female	2.0 (1.0, 8.0)	2.0 (1.0, 8.0)	0.14	0.893
Couple average	4.5 (1.5, 5.5)	4.25 (1.5, 5.0)	0.56	0.574
Couple difference (male minus female)	0.0 (− 2.0, 1.0)	0.0 (− 1.0, 1.0)	0.37	0.710

^a^Mann–Whitney test statistic

^b^Significant at p < 0.05 level

^c^Significant at p < 0.10 level

**Table 3:** Survival estimates and log-rank test results by couple average peer support score

Measure	Category	Estimate (95% CI)
Probability of outcome by nine months’ follow-up	4 points or less	0.65 (0.53, 0.75)
	5–6 points	0.61 (0.48, 0.72)
	7–10 points	0.80 (0.69, 0.87)
	11 points or more	0.78 (0.68, 0.85)
	Test statistic^a^	11.81
	df	3
	p-value	0.008

^a^Log-rank test statistic

An unadjusted bivariable discrete-time survival model indicated a significant association between couple average PS score and uptake (χ^2^ = 13.06, df = 3, p = 0.005), with uptake less likely amongst couples with average ≥ 7 points (OR 0.34, 95% CI 0.18, 0.68 [7–10 points]; OR 0.53, 95% CI 0.28, 0.99 [≥ 11 points]) compared to the reference category of ≤ 4 points (Table 4). In the final multivariable model adjusting for cohabitation, female-reported religion and couple difference PS score, the negative association between couple average PS score and uptake remained (AOR 0.59, 95% CI 0.25, 1.42 [7–10 points]; AOR 0.88, 95% CI 0.36, 2.10 [≥ 11 points]), but was no longer significant (χ^2^ = 6.55, df = 3, p = 0.088).

**Table 4:** Bivariable and multivariable associations between couple average peer support score and CHTC uptake

Variable	n (% with outcome)	Bivariable Model				Multivariable Model			
		OR (95% CI)	Test statistic^a^	df	p-value	Adjusted OR (95% CI)	Test statistic^a^	df	p-value
Couple average peer support score									
4 points or less	76 (34)	*Reference*	13.06	3	0.005^c^	*Reference*	6.55	3	0.088^d^
5–6 points	66 (38)	0.88 (0.48, 1.63)				1.44 (0.55, 3.75)			
7–10 points	88 (18)	0.34 (0.18, 0.68)				0.59 (0.25, 1.42)			
11 points or more	97 (22)	0.53 (0.28, 0.99)				0.88 (0.36, 2.10)			
Couple difference peer support score									
− 7 points or more (female higher)	67 (27)	0.73 (0.39, 1.39)	3.28	4	0.512	0.81 (0.32, 2.07)	1.14	4	0.887
− 6 to − 2 points	55 (27)	0.74 (0.38, 1.46)				0.93 (0.39, 2.18)			
− 1 to + 1 point	90 (31)	*Reference*				*Reference*			
+ 2 to + 6 points	57 (28)	0.83 (0.43, 1.62)				1.08 (0.44, 2.68)			
+ 7 points or more (male higher)	58 (19)	0.52 (0.25, 1.10)				0.70 (0.26, 1.89)			
Cohabitation									
No	251 (23)	*Reference*	13.93	1	< 0.001^b^	*Reference*	7.98	1	0.005^c^
Yes	76 (41)	2.64 (1.61, 4.32)				2.48 (1.42, 4.34)			
Religion (female reported)									
Christian	177 (24)	*Reference*	7.50	3	0.057^d^	*Reference*	8.79	3	0.032^c^
None	34 (47)	2.46 (1.28, 4.71)				2.56 (1.29, 5.07)			
Zionist	84 (27)	1.08 (0.63, 1.87)				0.97 (0.55, 1.72)			
Other	32 (22)	0.83 (0.36, 1.94)				0.67 (0.28, 1.63)			

N = 327 couples (all models)

All models were also adjusted for trial arm

^a^Test statistic from likelihood ratio test of additional explanatory variable

^b^ Significant at p < 0.001 level

^c^ Significant at p < 0.05 level

^d^ Significant at p < 0.10 level

When separately included in bivariable models adjusting for trial arm, male (χ^2^ = 5.74, df = 1, p = 0.017) and female (χ^2^ = 4.55, df = 1, p = 0.033) PS score were each significantly associated with uptake (Table 5). Although male and female variables used different category boundaries their estimated effect sizes were similar (male OR 0.58, 95% CI 0.37, 0.91 [≥ 8 points]; female OR 0.60, 95% CI 0.38, 0.97 [≥ 10 points]), and remained comparable (but no longer significant) when included together in a multivariable model (male χ^2^ = 1.61, df = 1, p = 0.205, AOR 0.73, 95% CI 0.45, 1.18 [≥ 8 points]; female χ^2^ = 1.54, df = 1, p = 0.215, AOR 0.73, 95% CI 0.44, 1.20 [≥ 10 points]).

**Table 5:** Bivariable and multivariable associations between partner-level peer support score and CHTC uptake

Variable	n (% with outcome)	Bivariable Model				Multivariable Model			
		OR (95% CI)	Test statistic ^a^	df	p-value	Adjusted OR (95% CI)	Test statistic ^a^	df	p-value
Male peer support score									
7 points or less	144 (33)	*Reference*	5.74	1	0.017^c^	*Reference*	1.61	1	0.205
8 points or more	183 (22)	0.58 (0.37, 0.91)				0.73 (0.45, 1.18)			
Female peer support score									
9 points or less	186 (31)	*Reference*	4.55	1	0.033^c^	*Reference*	1.54	1	0.215
10 points or more	141 (21)	0.60 (0.38, 0.97)				0.73 (0.44, 1.20)			
Cohabitation									
No	251 (23)	*Reference*	13.93	1	< 0.001^b^	*Reference*	8.68	1	0.003^c^
Yes	76 (41)	2.64 (1.61, 4.32)				2.39 (1.40, 4.08)			
Religion (female reported)									
Christian	177 (24)	*Reference*	7.50	3	0.057^d^	*Reference*	8.31	3	0.040^c^
None	34 (47)	2.46 (1.28, 4.71)				2.48 (1.28, 4.82)			
Zionist	84 (27)	1.08 (0.63, 1.87)				0.91 (0.52, 1.60)			
Other	32 (22)	0.83 (0.36, 1.94)				0.77 (0.32, 1.82)			

N = 327 couples (all models)

All models were also adjusted for trial arm

^a^Test statistic from likelihood ratio test of additional explanatory variable

^b^Significant at p < 0.001 level

^c^Significant at p < 0.05 level

^d^Significant at p < 0.10 level

## Discussion

We aimed to evaluate the association between couples’ perceived access to peer support and CHTC uptake in a high HIV prevalence area of South Africa. Many studies have described a social influence upon individuals’ uptake of HIV testing in SSA, with support from family, friends and peers frequently reported as an enabler and fears of stigmatisation and social exclusion as barriers [13]. Our findings point to a role in relation to couples contemplating testing together, suggesting that CHTC uptake by 9 months’ follow-up was less likely amongst couples with greater perceived access to peer support, conceptualised in terms of self-reported agreement with statements describing friendships outside of the primary partnership. In line with demographic trends in South Africa [45], most couples in our study cohort were unmarried and non-cohabiting, and cohabitation was a significant predictor of CHTC uptake in our bivariable and multivariable models. Previous qualitative research has suggested that non-cohabitation could contribute to worse intimacy and communication between partners in this setting [15], which may lead to relationships outside of the primary partnership becoming a more important source of personal support, giving greater prominence to normative attitudes around HIV testing and other health behaviours as expressed by friends and peers. Critically, and in contrast to testing programmes offered to individuals, the decision to participate in couples testing may result in knowledge that could be damaging to the relationship [7, 46], and must be taken jointly and accepted by both partners. Although CHTC has been proposed as a means of expanding testing, and reaching men in particular [47], men have tended to be less supportive of testing together than women [46, 48, 49]. The social context has been recognised as a structural influence on couples’ health and HIV risk behaviours [18] and, by acting independently and differentially upon each partner, could further constrain interactions and communication and exacerbate differences within the relationship, limiting capacity for behaviour change under the interdependence model [25].

The precise mechanism of influence is unclear owing to the composition of our independent variable of interest. Prioritisation of the couple average PS score could mask some aspects of individual partners’ contrasting exposures, although the finding that couple difference score was not a significant predictor in bivariable or multivariable models suggests that the magnitude of difference between partners was less important than the level of their average score. In addition, the meaning of the association is likely to be qualified by participants’ interpretation of the statements describing friendships outside of the primary partnership. For those assigning greater value to closer relationships, reported scores may describe exposure to the influence of a small number of trusted friends, whereas for those engaged with larger peer groups they may be more reflective of normative beliefs and attitudes circulating amongst peers. Nonetheless, our findings do suggest differing mechanisms of influence between the sexes. First, unadjusted comparisons by outcome indicated higher ‘friends’ and PS scores amongst males in couples without CHTC uptake, against little difference in female scores on any measure. Second, while male and female partner scores were significant predictors in separate bivariable models, binary categorisation at different boundaries indicated that males required a lower score to reach a similar couple-level effect to females. This may suggest that women were more influenced by primary relationship quality, while men tended to value peer group relationships more highly, and were more strongly influenced by them. This finding may align with previous research linking risk behaviours and attitudes to health-seeking to characteristics of male peer groups [29, 31, 50], and associating social norms more strongly with men’s, rather than women’s, willingness to test [51].

Couples-focused studies and interventions may be strengthened by considering the wider social context and addressing sources of support and influence outside of the primary partnership. Future research could explore the applicability of integrating existing peer support or social network measurement instruments into baseline couple assessments, or developing instruments to capture couple-specific social information, such as shared social contacts with potential to exert influence on both partners. An understanding of participating couples’ social context at recruitment could help to inform intervention design; they might, for example, seek to build positive social support structures from existing peer groups, or incorporate education or counselling components to help partners prioritise their relationship and build resilience in the face of harmful social influences [52]. Notably, some men are able to recast their conception of masculinity in terms emphasising social and familial responsibility and more compatible with health-seeking and preventive behaviours [28, 53], for example.

### Strengths and Limitations

This secondary analysis used data from a prospective study evaluating a couples-focused behavioural intervention; a key strength, therefore, was use of a dataset allowing for longitudinal analysis at individual partner and couple levels. Multivariable effect sizes were similar to those present in bivariable models but were no longer statistically significant; the cohort was large for a study of this nature, yet increased sample size would have improved the precision of estimates and may have confirmed multivariable findings as significant. Having defined our independent variable of interest using proxy measures capturing only one specific kind of social relationship our analysis can only provide a limited picture of social influence on this couples-focused outcome, but was designed as initial exploration of an area that, to our knowledge, has not yet been substantively addressed. A social network analysis examining couples’ overall network composition including the number, closeness and significance of relationships with friends, family members and other acquaintances, and individual and shared connections, may provide further clarity and nuance around the observed association. Self-reported PS scores may also be subject to social desirability bias if overstated to present a more favourable self-image or understated to emphasise dissatisfaction. Finally, voluntary participation in the primary study may have introduced self-selection bias if it attracted couples most willing to participate in such interventions, with potential to limit generalisability.

## Conclusions

Interventions designed to expand HIV testing and facilitate mutual disclosure amongst couples could be impactful in SSA, given the inherently dyadic dimension to transmission in this setting, but our findings suggest that, for some couples, social influences may create a structural barrier to uptake. The proximity and ubiquity of relationships and interactions within the lived social environment may mean that they resonate more strongly with individuals and couples in their capacity to influence, or discourage, behaviour change, presenting an important counterpoint to HIV prevention programmes. Accounting for that environment in the design of couples-focused interventions may increase their success, and could offer further opportunity to improve the wider appeal and acceptability of HIV prevention behaviours, contributing to a more healthful social context for all.

## Data Availability

The datasets generated during and/or analysed during the current study are available from the Principal Investigator (Lynae Darbes) on reasonable request, and also available upon request from the Human Sciences Research Council, South Africa.

## Abbreviations

HIV: Human immunodeficiency virus
SSA: Sub-Saharan Africa
CHTC: Couples HIV testing and counselling
PS: Peer support
OR: Odds ratio
AOR: Adjusted odds ratio
